# Construction of an IS-Free *Corynebacterium glutamicum* ATCC 13 032 Chassis Strain and Random Mutagenesis Using the Endogenous IS*Cg1* Transposase

**DOI:** 10.3389/fbioe.2021.751334

**Published:** 2021-12-15

**Authors:** Marten Linder, Markus Haak, Angela Botes, Jörn Kalinowski, Christian Rückert

**Affiliations:** ^1^ CeBiTec Bielefeld, Technology Platform Genomics, Bielefeld University, Bielefeld, Germany; ^2^ Department of Biology, Massachusetts Institute of Technology, Cambridge, MA, United States; ^3^ School of Molecular and Cell Biology, University of the Witwatersrand, Johannesburg, South Africa

**Keywords:** corynebacterium glutamcium, IS elements, prophages, genetic switch, “genome healing”, mutagenesis, ISCg1

## Abstract

Mobile genetic elements (MGEs) contribute to instability of the host genome and plasmids. Previously, removal of the prophages in the industrial amino acid producer *Corynebacterium glutamicum* ATCC 13 032 resulted in strain MB001 which showed better survival under stress conditions and increased transformability. Still, eight families of Insertion Sequence (IS) elements with 27 potentially active members remain in MB001, two of which were demonstrated to be detrimental in biotechnological processes. In this study, systematical deletion of all complete IS elements in MB001 resulted in the MGE-free strain CR101. CR101 shows growth characteristics identical to the wildtype and the increased transformability of MB001. Due to its improved genome stability, we consider this strain to be an optimal host for basic research and biotechnology. As a “zero-background” host, it is also an ideal basis to study *C. glutamicum* IS elements. Re-sequencing of CR101 revealed that only five spontaneous point mutations had occurred during the construction process, highlighting the low mutation rate of *C. glutamicum* on the nucleotide level. In a second step, we developed an easily applicable IS*Cg1*-based transposon mutagenesis system to randomly transpose a selectable marker. For optimal plasmid stability during cloning in *Escherichia coli*, the system utilizes a genetic switch based on the phage integrase Bxb1. Use of this integrase revealed the presence of a functional *attB* site in the *C. glutamicum* genome. To avoid cross-talk with our system and increase ease-of-use, we removed the *attB* site and also inserted the Bxb1 encoding gene into the chromosome of CR101. Successful insertion of single markers was verified by sequencing randomly selected mutants. Sequencing pooled mutant libraries revealed only a weak target site specificity, seemingly random distribution of insertion sites and no general strand bias. The resulting strain, ML103, together with plasmid pML10 provides a easily customizable system for random mutagenesis in an otherwise genomically stable *C. glutamicum*. Taken together, the MGE-free *C. glutamicum* strain CR101, the derivative ML103, and the plasmid pML10 provide a useful set of tools to study *C. glutamicum* in the future.

## 1 Introduction

*Corynebacterium glutamicum* is a GRAS-classified (Generally Recognized as Safe), Gram-positive bacterium which is known to be non-pathogenic, non-motile, and non-sporulating. Due to its safety of use, its easy genetic accessibility, and good performance parameters (fast growth on cheap carbon sources, resistance to changes in pO2, etc.), it is widely used in fermentation processes today (Becker and Wittmann, 2012). Besides production of amino acids and other bulk chemicals (Wendisch, 2020, 2016), it is also applied for production of high-value compounds (Wolf et al., 2021). While many *C. glutamicum* strains have been isolated, to our knowledge just two are widely used in basic research and biotechnology: strain ATCC 13 032 and strain R. This might in part be due to the presence of the paracrystalline protein surface (S) layer in many other *C. glutamicum* strains (Hansmeier et al., 2004) that was shown to interfere with DNA transfer by electroporation in *Caulobatcer* species (Gilchrist and Smit, 1991) and in part due to the early availability of complete genome sequences for the two strains (Ikeda and Nakagawa, 2003; Kalinowski et al., 2003; Yukawa et al., 2007). Of those two, strain ATCC 13 032 not only constitutes the type strain of the species, but is used by most researchers, while strain R is apparently used exclusively by researchers at the Research Institute of Innovative Technology for the Earth (Kyoto, Japan).

While *C. glutamicum* has many beneficial properties that make it well suited for industrial applications and basic research, it still suffers from the problem of all living systems, i.e., the spontaneous occurrence of mutations. Fortunately, the genomes of *Corynebacterium* species are known to be very stable on the large scale, with even distant species sharing a high degree of chromosomal synteny (Ikeda and Nakagawa, 2003; Yukawa et al., 2007) which is attributed to the lack of a complete recombination system (Nakamura et al., 2003). In contrast to this stability on the large scale, the genome of *C. glutamicum* contains a large number of mobile genetic elements (MGEs) (Kalinowski et al., 2003; Yukawa et al., 2007) which might cause instability on the gene level. Indeed, removal of MGEs and prophages was shown to greatly enhance the stability of the genome (Umenhoffer et al., 2010; Renda et al., 2014). This has been demonstrated conclusively for the model and production organisms *Bacillus subtilis* (Ozaki et al., 2007) and *Escherichia coli* (Pósfai et al., 2006; Umenhoffer et al., 2010). For *C. glutamicum*, there is good evidence that at least some of the prophages and IS elements present are active and can affect the behaviour of the bacteria. For example, Frunzke et al. (2008) showed that the prophage CPG3 found in strain ATCC 13 032 can excise from the genome, replicate as an episome and cause cell lysis when present in high copy numbers. Furthermore, the available genome sequences for *C. glutamicum* ATCC 13 032 differ in the the number of prophages and IS elements present (Kalinowski, 2005), another indication of at least some of them being active.

Concerning the creation of an MGE-free *C. glutamicum* strain, some prior work exists, but no rigorous effort to remove all potentially active MGEs has been reported so far. For example, Suzuki et al. (2005) removed a total of eight regions in strain R, comprising 190  kbp (188 genes) which contained, among others, one prophage and 17 IS elements, and demonstrated that the resulting strain displayed wildtype-like growth. Regarding the removal of IS elements, Choi et al. (2015) created two strains, one lacking all four copies of IS*Cg1* (WJ004) and one lacking all four copies of IS*Cg2* (WJ008). Interestingly, they found that their version of strain ATCC 13 032 contained only four copies of IS*Cg2* instead of the five reported by Ikeda and Nakagawa (2003) and Kalinowski et al. (2003) and that one copy of IS*Cg1*, IS*Cg1c*, was absent and replaced by a copy at a different position. For both strains, they observed improvements in heterologous protein expression as well as transformability. Last but not least, Baumgart et al. (2013) removed the three prophages CGP1-CPG3 present in their version of strain ATCC 13 032. The resulting strain, MB001, also displayed wildtype-like growth but increased transformability and improved expression of a heterologous protein due to an increased copy-number of the used vector. Based on these findings, we therefore build upon previous works (Baumgart et al., 2013) to establish an MGE-free strain by removing all potentially active IS elements in the prophage-free strain MB001. The version of strain ATCC 13 032 sequenced in Bielefeld contains a total of 38 potential IS elements (27 considered to be complete, 11 partials) belonging to eight families (Kalinowski et al., 2003). While the IS*L3* and IS*30* families have only one member each in this strain, IS*Cg1* (4 copies) and IS*Cg2* (5 copies), the IS*3* family is by far the most abundant and diverse (9 instances from five members). With one of its members, IS*Cg14*, being located on the deleted prophage CGP3, this leaves 26 elements to be removed in strain MB001.

Besides being an optimal host for basic research and biotechnology due to its improved stability, an MGE-free strain also provides an ideal basis to study native IS elements as well as to establish and test random mutagenesis systems based on these. In combination with high-throughput sequencing such IS-based systems have a wide range of applications (Cain et al., 2020). For example, Suzuki et al. (2006) used IS*Cg1* in strain R which naturally lacks this IS element to identify essential genes in *C. glutamicum*. Likewise, mutagenesis by larger elements can be helpful in adaptive laboratory evolution experiments, as this allows not only for gene disruption but also for gene activation (Hennig et al., 2020). Therefore, we endeavored to build an IS*Cg1*-based random mutagenesis system that allows for, among others, controllable random insertion of a selectable marker. To facilitate cloning and replication of transposition constructs in a host such as *E. coli*, we used a genetic switch based on a genomically integrated Bxb1 phage integrase (Bonnet et al., 2012), activating expression only after transfer to *C. glutamicum*.

The strains created in this study should not only aid basic research by combining generally wildtype-like behaviour with reduced random mutation events, but also industrial applications as IS elements contribute to the degeneration of bacterial production strains (Peng and Liang, 2020). Furthermore, IS*Cg1*-based random mutagenesis allows for multi-copy insertion of genes of interest, a method that was shown to significantly increase, e.g., heterologous protein expression (Yomantas et al., 2011).

## 2 Materials and Methods

### 2.1 Bacterial Strains, Plasmids and Culture Media

The bacterial strains and plasmids used in this study are listed in Table 1 and Supplementary. Table S2, respectively. Plasmids pCRn100 days to pCRn125 days, pMLi002 and pMLd009 are based on the pK18 *mobsacB*  vector (Schäfer et al., 1994a). pCRn100 days to pCRn125 days were used for successive genomic deletion of IS*Cg* elements in MB001. pMLi002 allows for genomic integration of the coding sequence of the Bxb1 phage integrase at the former locus of IS*Cg3a* deleted by pCRn110 days pMLd009 is used to remove *groEL*1 in the genome. The plasmid pML10 was build using pUC19 as backbone. It is used for cloning and replication in *E. coli* and as a transposition construct in *C. glutamicum*. It carries a kanamycin resistance cassette flanked by the IS*Cg1* inverted repeats as transposable element as well as the transposase encoded by *tnp1*. Expression of this gene in turn depends on a Bxb1 integrase-based switch (Bonnet et al., 2012). *E. coli* strains carrying plasmids were routinely grown on solid Antibiotic Medium No. 3 (PA) (Oxoid, Wesel, Germany) at 37°C. *C. glutamicum* strains were grown on solid brain-heart broth (BH) (VWR International, Darmstadt, Germany) at 30°C. Antibiotics used for selection of plasmids and strains were nalidixic acid (50 *μ*g/ml for corynebacteria) and kanamycin (50 *μ*g/ml for *E. coli*, 25 *μ*g/ml for corynebacteria).

**TABLE 1:** Bacterial strains.

Name	Relevant genotype/information ^*a*^	Source/References
*E. coli* DH5*α*MCR	F^−^ *endA1 supE44 mcrA thi-1 hsdR17 λ* ^−^ *recA1 relA1* Δ(*lacZYA-argF*) *U169* (Φ80d*lacZ*Δ *M15*) *gyrA96 deoR* Δ(*mrr-hsdRMS-mcrBC*)	Grant et al. (1990)
*C. glutamicum*		
ATCC 13 032	Wild type, Nx^r^	ATCC ^*b*^
ATCC 13 869	“*Brevibacterium lactofermentum*”, Nx^r^	ATCC
ATCC 14 067	“*Brevibacterium flavum*”, Nx^r^	ATCC
AS 1.542	“*Corynebacterium crenatum*”, Nx^r^	Chen et al. (2012)
MB001	*C. glutamicum* ATCC 13 032 with deleted prophages: ΔCGP1, ΔCGP2, and ΔCGP3	Baumgart et al. (2013)
CR099	*C. glutamicum* MB001 with deleted IS elements ΔIS*Cg1a*, ΔIS*Cg1b*, ΔIS*Cg1c*, ΔIS*Cg1d*, ΔIS*Cg1e*, ΔIS*Cg2b*, ΔIS*Cg2c*, ΔIS*Cg2d*, ΔIS*Cg2e*, and ΔIS*Cg2f*	Baumgart et al. (2018), this study
CR100	*C. glutamicum* CR099 with deleted IS elements ΔIS*Cg5a*, ΔIS*Cg5b*, ΔIS*Cg5c*, ΔIS*Cg8*, ΔIS*Cg12*, ΔIS*Cg13a*-ΔIS*Cg21a*, ΔIS*Cg13b*, ΔIS*Cg16a* and ΔIS*Cg16b*	this study
CR101	*C. glutamicum* CR100 with deleted IS elements ΔIS*Cg3a*, ΔIS*Cg3b*, ΔIS*Cg4*, ΔIS*Cg6a*-ΔIS*Cg7*, ΔIS*Cg6c*, ΔIS*Cg9*, and ΔIS*Cg15a-b*	this study
ML102	CR101 with *bxbI* inserted at ΔIS*Cg3a*	this study
ML103	ML102 with Δ*groEL*	this study

^*a*^ r superscript indicates resistance. Nx, Nalidixic acid; Km, Kanamycin

^*b*^ ATCC; American Type Culture Collection, Rockville, MD

### 2.2 DNA Isolation, Transfer and Manipulation

Standard procedures were employed for molecular cloning and transformation of *E. coli* DH5*α*, as well as for electrophoresis (Sambrook et al., 1989). Transformation of *C. glutamicum* was performed by electroporation using the methods of Tauch et al. (2002).

Sequence similarity-based searches with nucleotide sequences were performed using the basic local alignment search tool (BLAST, Altschul et al., 1990).

### 2.3 Construction of Plasmids

Plasmids pCRn100 days to pCR125 days as well as pMLi002, pMLd009 and pML10 were constructed using Gibson assembly (Gibson et al., 2009). The primers used are listed in Supplementary. Table S1. In case of “genome healing” constructs, the final insert was amplified directly from either “*Brevibacterium lactofermentum*” ATCC 13 869, “*Brevibacterium flavum*” ATCC 14 067, or “*Corynebacterium crenatum*” AS 1.451 gDNA using the respective primers _d1 and _d4. In all other cases, two products (primer pairs _d1 and _d2 respectively _d3 and _d4) were amplified from *C. glutamicum* MB001 gDNA. The insert(s) were then assembled with linearized pK18 *mobsacB* amplified via the primers pK18_ga1 and pK18_ga2. Primers pk18_dgroel1 and pk18_dgroel2 were used to linearize pK18 *mobsacB* for *groEL*1 deletion. The homologous flanks were amplified with the primers d1_groel1-d4_groel1 from ATCC 13 032. Linearization of pCRn110 days for insertion of the Bxb1 integrase coding sequence was achieved with the primer pair pK18_bxbI_1 and pK18_bxbI_2 and the sequence of the Bxb1 integrase was amplified with the primer pair bxbI_ins1 and bxbI_ins2 from *Mycobacterium smegmatis*. The backbone for pML10 was linearized with the primer pair switch_ga1 and switch_ga2 from pUC19. The switch, consisting of the Bxb1 integrase *attB* and *attP* flanking the original constitutive Anderson promoter (Anderson, 2013) was amplified via the overlapping primers *attP*_Pcons and Pcons_*attB*. *Kan* ^r^ originates form pK18 *mobsacB* and the imperfect inverted repeats for IS*Cg1* are from ATCC 13 032. All PCRs were performed using Phusion High–Fidelity DNA polymerase, the assembly was done using Gibson Assembly Master Mix (both Thermo Fisher Scientific, Germany). The assembly mixture was used to transform *E. coli* DH5*α*MCR, the transformants were selected on PA plates containing 50 *μ*g/ml kanamycin and 40 mg/L X-Gal (5-bromo-4-chloro-3-indolyl-*β*-d-galactopyranoside) and verified by Sanger sequencing.

### 2.4 Site-specific Gene Disruption/Replacement

Site-specific gene disruption was performed using the non-replicable integration vector pK18mobsacB which allows for marker-free deletion of the target gene (Schäfer et al., 1994c). The resulting plasmids pCR100 days to pCR124 days and pMLi002and pMLd009 were transformed into *C. glutamicum* MB001 respectively subsequent deletion mutants by electroporation (Tauch et al., 2002). Integration of the introduced plasmids into the chromosome by single-crossover was tested by selection on BH plates containing 25 *μ*g/ml kanamycin. For the deletion of the target gene, the kanamycin-resistant (Km^r^) cells were grown overnight in liquid BH and spread on BH plates containing 10% sucrose. Cells growing on this plate were tested for kanamycin sensitivity (Km^s^) by parallel picking on BH plates containing either kanamycin or sucrose. Sucrose-resistant and kanamycin-sensitive cells were then tested for the deletion by PCR and checked for IS activity using Southern blotting and hybridization (Evans et al., 1994) if deemed appropriate.

### 2.5 Transposon Mutant Sequencing

Genomic DNA was isolated using the NucleoSpin Microbial DNA Mini kit (Macherey-Nagel, Germany). Single mutant colonies were sequenced using the Rapid Barcoding Kit SQK-RBK004 (Oxford Nanopore Technologies, UK) on R9.4.1 flowcells on a GridION.

For sequencing of complete mutant libraries, all colonies were pooled before genomic DNA was isolated. Subsequently, the gDNA was fragmented to approximately 10,000  bp fragments using a Covaris g-TUBE. The ends of the fragments blunted and an A-overhang was added with the NEBNext Ultra II End Repair/dA-Tailing Module. The A-overhang is then used for ligation of dsDNA adapters derived from annealing the oligos D7 and D7_top carrying part of the TruSeq D7 sequence. In a first round of PCR using Phusion High–Fidelity DNA polymerase, fragments are amplified between a biotinylated primer tnp_o, binding in the kanamycin resistance marker about 500 nt upstream of the inverted repeat, and the TruSeq D7 primer. After amplification, fragments were purified with Dynabeads M-270 Streptavidin binding the biotinylated fragment ends. During washing of the trapped DNA on a magnet rack, 0.1 mM NaOH was applied to retain ssDNA of the amplified region. This is followed by a half-nested second round of PCR with LongAmp Taq DNA Polymerase. Here, primer D7 is paired with a phosphorylated nested primer tnp_i, located 150 bp from the 3′-end of the kanamycin resistance cassette.

After purification, the DNA was used in the adapter ligation step of the Ligation Sequencing Kit SQK-LSK109 (Oxford Nanopore Technologies) and sequenced on an R9.4.1 flowcell on a GridION. The sequencing reads where mapped to pML10 and *C. glutamicum* CR101 respectively using minimap2 (Li, 2018) with options-cx map-ont–eqx–secondary = no. Reads mapping to both references were processed using custom Python scripts to identify transposition sites based on the local alignments. These were visualized in a polar genome plot using the Python library Matplotlib (Hunter, 2007). Sites with at least two mapped reads were subjected to an analysis of the target site of IS*Cg1* by creating a sequence logo with WebLogo3 (Crooks, 2004) based on the strand-specific 8 bp of genomic sequence downstream the inverted repeat at each transposition site.

### 2.6 Determination of Growth Rates and Transformation Efficiency

Cultivations to check for differences in growth behaviour of MB001, CR101, ML102 and ML103 were performed using a BioLector I (m2p-labs). Four biological replicates of each strain were cultivated in four technical replicates. Samples were cultivated in 1 ml BHI at 30°C, 1,100 rpm, and at 85% humidity. Measurements were taken every 15 min and the growth rates were calculated for each sample with the BioLection software using a sliding window of eight data points (i.e. 2 h). Afterwards, for each strain first the average growth rate for each biological replicate was calculated, followed by calculation of the mean and standard deviation from those averages.

The transformation efficiency was determined as described by Baumgart et al. (2013) with one modification: In case of the integrative plasmid, 1,000 ng instead of 500 ng were used. For each strain, three independent cultivations, i.e. biological replicates, were performed to create competent cells as described by Tauch et al. (2002). From each batch prepared in that manner, four aliquots were used for electroporation with the respective plasmid, serving as technical replicates.

### 2.7 Genome Sequencing, Assembly, and Accession Numbers

The complete genome sequence of *C. glutamicum* CR101 was determined using a hybrid assembly approach as described by Ballas et al. (2020). All sequences from other *C. glutamicum* strains were obtained from GenBank (Agarwala et al., 2017): BX927147 (*C. glutamicum* ATCC 13 032), CP005959 (*C. glutamicum* MB001), CP016335 (“*B. lactofermentum*” ATCC 13 869), CP022614 (“*B. flavum*” ATCC 14 067), and LOQT01 (“*C. crenatum*” AS1.452). All data generated during this project is available *via* BioProject PRJNA750341.

## 3 Results

### 3.1 Construction of Strain CR101

To construct strain CR101, containing no potentially active IS elements (Kalinowski et al., 2003; Pfeifer-Sancar et al., 2013), strain MB001 lacking all prophages (Baumgart et al., 2013) was used as a basis. Interestingly, the genome of this strain contains five copies of IS*Cg1*, named IS*Cg1e*, which is located at position 541,217 to 542,527 (CP005959) and disrupts gene *cgp*_*0611*. To determine the boundaries of the necessary deletions as well as to amplify the inserts used for double-crossovers, we applied a “genome healing” approach: First, using BLAST we searched for the sequence of the IS*Cg* elements with 2,500 bp flanks in the genome sequences of 3 *C. glutamicum* strains available at our lab (“*Brevibacterium lactofermentum*” ATCC 13 869, “*Brevibacterium flavum*” ATCC 14 067, and “*Corynebacterium crenatum*” AS1.452) to identify the orthologous regions in the three genomes. In those cases where 1) there was a syntenous region present, but 2) without the IS*Cg* element found in MB001, we then compared the number of differences between the target sequence and that of MB001 in the 750 bp directly up- and downstream of the missing region. If these showed >99% identity, primers were designed to amplify a deletion construct with 500–600 bp flanking sequences. In case of more than one potential candidate, i.e., a region satisfying the rules present in more than one genome, the candidate with the highest similarity to the region in MB001 was selected. Using this approach, 12 regions were identified in ATCC 13 869, three in ATCC 14 067, and two in AS1.452 (see Supplementary Table  S2). For IS*Cg1e*, a fifth copy of IS*Cg1* that was absent in the ATCC 13 032 wildtype (Kalinowski et al., 2003, Table two), the region was compared to the wildtype genome and genomic DNA of the wildtype was used to amplify the construct for “genome healing”. For the remaining eight regions, deletion constructs were build conventionally, based on RNAseq data (Pfeifer-Sancar et al., 2013) to remove the CDS as well as the corresponding promoter.

These constructs were then used to sequentially remove the IS elements, starting with IS*Cg1* and IS*Cg2* as the supposedly most active. This resulted in strain CR099 (see Supplementary Figure  S2) which was already successfully used as the basis in other genome reduction projects (Baumgart et al., 2018). To ensure that no further jumps of IS elements still remaining in the respective strain had occurred, the presence of the various IS elements was checked by Southern blotting and hybridization at key points, usually at least after removal of all instances of an element present in several copies or after deletion of three to four copies of elements present only once. Thereby, we were able to detect a total of seven jumps of IS*Cg1* (see Supplementary Figure S1 for an example), two jumps of IS*Cg2*, one of IS*Cg5*, one of IS*Cg13* and one of IS*Cg16* (data not shown). The strain with those active as well as several other elements removed was dubbed CR100 (see Supplementary  Figure S2). Removal of the remaining seven elements progressed without further complications, resulting in the final, prophage- and IS-free strain CR101.

To ensure the complete removal as well as to detect all other mutations introduced during the 26 rounds of genetic manipulation, the complete genome sequence of *C. glutamicum* CR101 was established using a hybrid assembly approach based on Illumina short read and Oxford Nanopore Technologies (ONT) long read data. The final genome sequence has a size of 3,034,563 bp, compared to 3,079,25 bp for MB001. Comparison of the annotated genomes revealed that 11 genes were “healed” through the removal of the various IS elements. While a large number of synonymous and missense mutations was introduced in the flanking regions, they all corresponded to the sequences found in the donor genomes and are thus unlikely to change the function of genes encoded in these regions. On the other hand, only four missense mutations and one frameshift were detected in the remainder of the genome, indicating an overall low rate of spontaneous mutations in *C. glutamicum*. The final strains were also checked for changes in growth rate and transformability. Strains CR101, ML102, and ML103 showed no significant difference in growth rate compared to the progenitor *C. glutamicum* MB001 (Supplementary  Table  S3). In case of transformability, a slight increase in the number of transformants per *μ*g DNA could be observed (Supplementary  Figure S3), but analysis via two-way ANOVA with replication indicates that this is not significant in case of the replicative plasmid (*p* = 0.44 for an alpha of 0.05) as the variance between biological replicates far exceeds that among the four strains. In case of the integrative plasmid, the observed change is statistically significant (*p* = 0.023), but again the variability between batches of competent cells is extremely high.

### 3.2 Development of a Genetic Switch Based System for Random Insertion Mutagenesis

As random insertion mutagenesis is of use in some experimental contexts, e.g., adaptive laboratory evolution Hennig et al. (ALE 2020), we decided to re-purpose IS*Cg1*, using a system similar to that developed by Suzuki et al. (2006).

**FIGURE 1:**
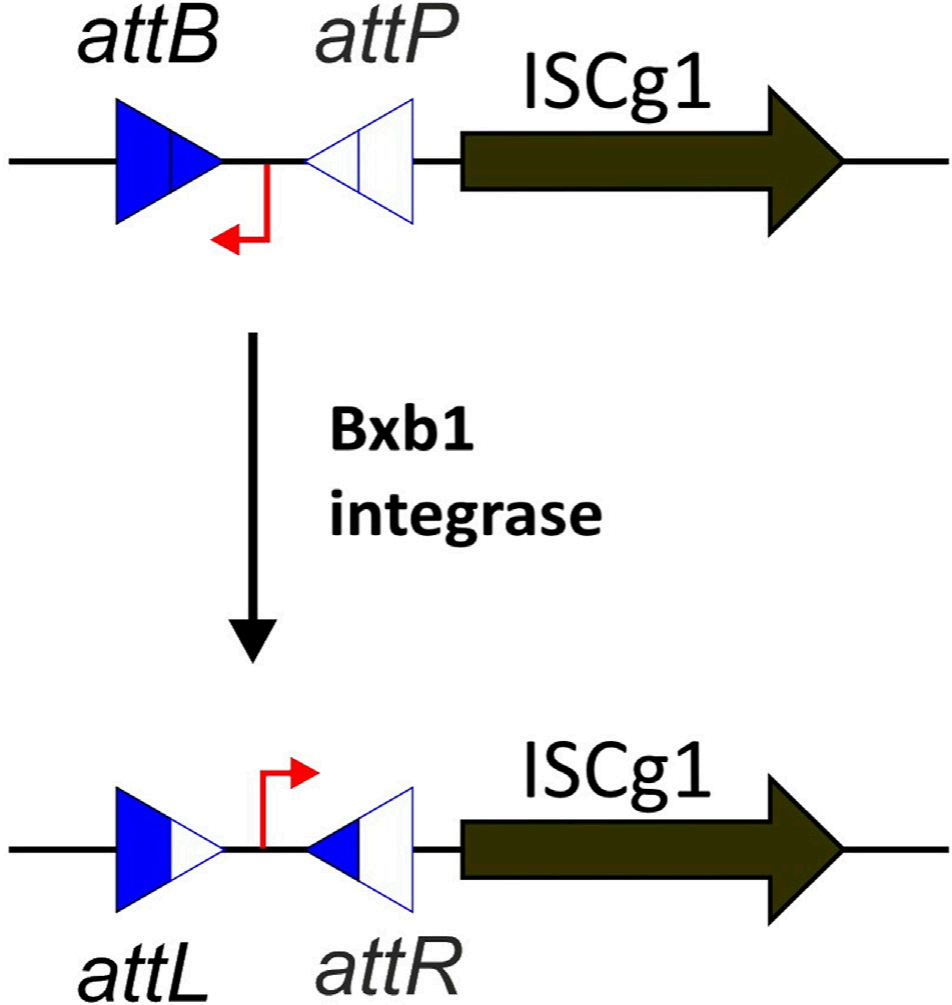
Principle of the Bxb1 phage integrase genetic switch. In its default state, the promoter (in red) is in opposite orientation to the IS*Cg1* transposase and therefore no expression takes place. As soon as the integrase is abundant, the bacterial and phage attachment sites *attB* and *attP* are targeted by the integrase, transitioning the switch into the active state by recombination, forming the hybrid attachment sites *attL* and *attR*.

In most scenarios, only a single transposition event per mutant is desired. Therefore, pUC19 was used as a transposition construct, as it is a non-replicating vector in *C. glutamicum* and allows for easy replication and isolation in *E. coli* (Yanisch-Perron et al., 1985). We then used the regular IS*Cg1* sequence from ATCC 13 032 as transposase and a kanamycin resistance cassette flanked by the 24 bp imperfect terminal inverted repeats as transposon. This way, only a successful transposition into the genome provides resistance to kanamycin. However, problems occurred when we tried to isolate the plasmid from *E. coli* because IS*Cg1* transposition turned out to be highly active in *E. coli* already. This resulted in mostly “empty” plasmids, i.e., plasmids from which the transposon cassette had jumped into the *E. coli* chromosome. To acquire sufficient quantities of active plasmid for large-scale transposon mutagenesis, the plasmid pML10 was designed using a Bxb1 phage integrase genetic switch inserted upstream of IS*Cg1* (Figure 1). The switch consists of a constitutive promoter flanked by the bacterial and phage attachment sites targeted by the Bxb1 integrase (Bonnet et al., 2012). By default, the promoter is in opposing orientation to IS*Cg1* such that no expression is occurring and subsequently no transposition takes place. To invert the switch and enable expression of IS*Cg1*, the coding sequence for the Bxb1 integrase has been inserted into the genome of CR101 at the former locus of IS*Cg3a* creating ML102. As soon as pML10 is transformed into ML102 the Bxb1 integrase targets the *attB* and *attP* attachment sites flanking the promoter, forming the hybrid attachment sites *attL* and *attR* by recombination. In the process, the orientation of the promoter is inverted and drives the expression of IS*Cg1*.

**FIGURE 2:**
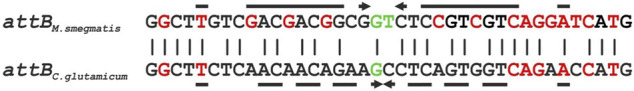
Alignment of the minimal required bacterial attachment site sequences of the phage Bxb1 in its natural host *M. smegmatis* and the observed attachment site in *C. glutamicum*. Base pairs coloured in red are specific for *attB* identity in *M. smegmatis*. The green central GT is the essential dinucleotide for recombination and the dashed arrows indicate palindromic base pairs.

During sequencing of the first mutants, we noticed the occurrence of integration of the whole transposition construct pML10 into *groEL*1 with high frequency (data not shown). Examination of the sequences of several mutants revealed that all these insertions occurred at the same site and with the same directionality. The subsequent comparison of the bacterial attachment site in *M. smegmatis* to the integration site of our mutants showed a significant degree of similarity, indicating a partially conserved *attB* site in *groEL*1. Recognition of this site by the Bxb1 integrase then results in integration of the plasmid via the *attP* site of the genetic switch. Interestingly, many nucleotides defining *attB* functionality as well as the central GT dinucleotide thought to be essential (Singh et al., 2013) are not well conserved in the site present in *C. glutamicum groEL*1 (Figure 2).

To circumvent the genomic integration via the Bxb1 integrase as a competing event to transposition via IS*Cg1*, we deleted *groEL*1. While this gene is essential in many organisms, there are two copies present in *C. glutamicum* ATCC 13 032 and *groEL*1 was actually disrupted by IS*Cg1c* prior to its removal in CR099 and thus CR101. In subsequent mutant libraries of the strain ML103 no further display of integration via Bxb1 integrase was observed.

### 3.3 Sequencing of ML103 Transposon Mutants

To test our genetic-switch-based system for random insertion mutagenesis, 200 transformations via electroporation of 800 ng pML10 each into ML103 were performed. On most agar plates less than 0.5 ⋅ 10^2^ colonies were observed, but several cases with hundreds of colonies were also detected. From the resulting transposon mutants generated in ML103, we selected 12 single colonies for whole genome sequencing. One mutant colony was picked because of its noticeably intense yellow phenotype (Supplementary  Figure  S4), the others were selected randomly. In each of these mutants, only one transposition took place, each located at a different genomic position. The complete genome sequence of the phenotypically distinct mutant revealed a transposition into *crtR* which has been characterized as encoding a MarR-type repressor. CrtR represses the *crt* operon which is involved in decaprenoxanthin synthesis, a yellow carotenoid. Deletion of *crtR* increases transcription of the *crt* operon by 10 to 30-fold (Henke et al., 2017), probably explaining the striking phenotype of our mutant.

**FIGURE 3:**
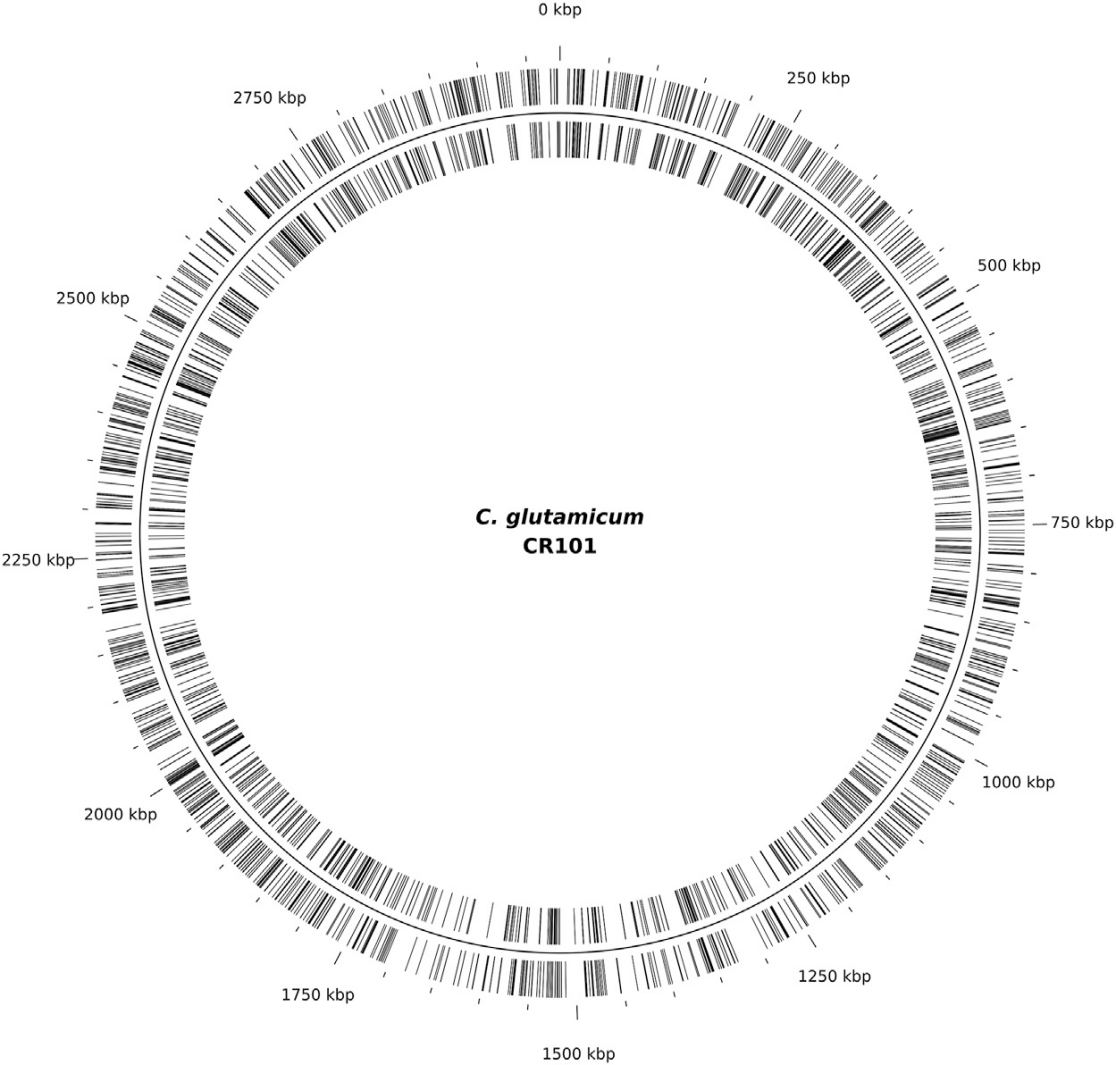
Transposition sites mapped to the genome of CR101. A total of 3,207 transposition sites were identified by mapping sequencing reads of the random mutagenesis experiment performed with *C. glutamicum* ML103 to *C. glutamicum* CR101, with 1,582 sites on the leading strand (outer lines) and 1,625 sites on the lagging strand (inner lines).

**FIGURE 4:**
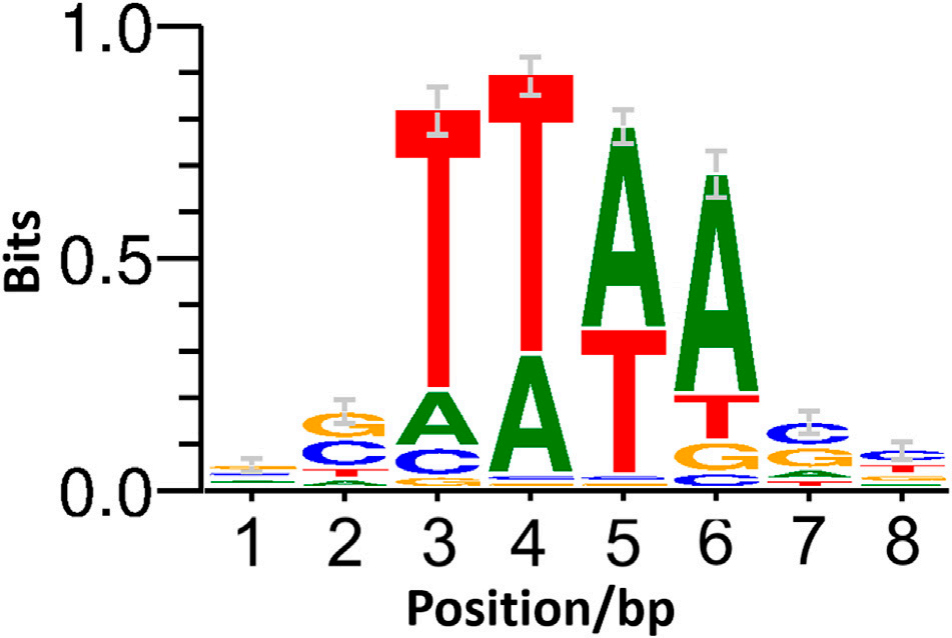
Weblogo of the IS*Cg1* target site. WebLogo (Crooks, 2004) of the IS*Cg1* target site based on the strand-specific 8 bp of genomic sequence downstream of the inverted repeat at transposition sites with at least two mapped reads.

To further determine the suitability of IS*Cg1* in terms of target site bias, all mutants were pooled and ligation-based ONT sequencing libraries were constructed by targeted enrichment of the region downstream of the selection marker. Sequencing resulted in 3,207 independent transposition sites indicating no obvious target site preference on a genomic scale (Figure 3). Allocation of transposition sites with respect to strand directionality is evenly distributed with 1,582 sites on the leading strand and 1,625 sites on the lagging strand. Weblogo analysis of the eight bp target site of IS*Cg1* (Figure 4) suggests a bias towards loci with high AT content in the central four base pairs with strong discrimination against G and C at position four and 5. Frequency analysis of the central tetranucleotide (Supplementary Table S4) shows that no specific single sequence appears to be predominant which is in line with previous analyses (Inui et al., 2005).

## 4 Discussion

The first goal of this work, the construction of a prophage- and IS-free *C. glutamicum* strain derived from the industrially relevant type strain ATCC 13 032, was successfully achieved. While no obvious differences compared to the progenitor strain MB001 could be observed, this result was predictable and is actually the favored outcome. Like Choi et al. (2015) who reported a significant increase in transformability in strains lacking all copies of either IS*Cg1* or IS*Cg2*, we also observed a slight increase, albeit not significant in our case. This is unsurprising as there is no molecular basis for this behaviour: none of the genes restored is known or suspected to play a role in DNA uptake or any other associated function. The most likely explanation for this discrepancy is the progenitor strain used for the removal of the IS elements (see Supplementry  Figure S3): Choi et al. (2015) used the wildtype ATCC 13 032 while we used the derivative MB001, already lacking the prophages (Baumgart et al., 2013). Interestingly, MB001 shows properties akin to those reported by Choi et al. (2015). As at least one of the prophages, CGP3 which also carries a restriction-modification system known to be detrimental to plasmid uptake in *C. glutamicum* (Schäfer et al., 1994b), was shown to be unstable and prone to spontaneous excision and maybe loss (Frunzke et al., 2008), it stands to argue that such an event happened during the works of Choi et al. (2015). Since various rounds of plasmid-dependent deletions were used, this would actually be positively selected for. Likewise, the unchanged growth rate was expected, as again this was the intended outcome. Indeed, even when combining 11 large scale deletions in strain CR099, totaling about 240 kbp, Baumgart et al. (2018) observed no detrimental effect on the growth rate of the mutant when compared to the wildtype.

Meanwhile, the work of Choi et al. (2015) as well as ours showed that at least some IS elements in *C. glutamicum* are highly active, especially IS*Cg1*. For example, the heterogeneity of TAG-producing cells (Fig. 6 Plassmeier et al., 2016) was later found to be caused by IS*Cg1* jumping into the production plasmid (C. Rückert, personal observation, 2017). Thus, *C. glutamicum* CR101 should be an ideal host to profit from increased stability while retaining wildtype behaviour in almost all other aspects.

Yet, while genome stability is preferable in most situations in basic research and industrial biotechnology, the availability of an reliable mutagenesis system with one transposition event per mutant is also a valuable tool. Therefore, we decided to use IS*Cg1* due to its proven activity in *C. glutamicum* and demonstrated value for whole genome analysis (Suzuki et al., 2006). Surprisingly, this approach initially resulted in a failure caused by IS*Cg1* activity during plasmid propagation in *E. coli*. While again delivering proof of high transposition activity mediated by IS*Cg1* transposase, this made direct use of our system difficult. It is interesting to note that, to the best of our knowledge, these problems have not been reported before as this problem should occur with any transposon that is active in *E. coli*. To solve this problem, we considered using a repressor-based system, but such circuits are often challenging due to minimal expression leakage (Kato, 2020) and often require inducer substances to be effective. Therefore, we turned to the Bxb1 integrase which is highly directional with no need for additional cofactors or proteins (Kim et al., 2003) to create a system that is silent in *E. coli* and only becomes active when transferred to the proper *C. glutamicum* strain expressing Bxb1. Indeed we observed no activity of pML10 in *E. coli*. Sequencing of randomly selected single transposon mutants in ML103 revealed only single transposition events, validating the design concept.

In the scope of this work we also observed an intense yellow-coloured mutant colony which sequencing identified as an integration of the transposon into *crtR*, characterized by Henke et al., 2017 as repressor of the *crt* operon. In their work, deletion of *crtR* increased decaprenoxanthin synthesis by 10–30 fold compared to the wildtype (Henke et al., 2017). This indicates the potential of the system to also screen for phenotypically striking mutations.

Also of potential interest is the integration of pML10 into a partially conserved *attB* site in *groEL*1. The GroEL gene is the target for Bxb1 prophage integration in its natural host *M. smegmatis* (Kim et al., 2003). Using *C. glutamicum* ML102, this allows for targeted integration of any vector carrying the proper *attP* site.

One problem that needs to be addressed is the fairly low efficiency of our system. The observation of several electroporation events giving rise to more than a thousand colonies demonstrates that the system works in principle but is constrained by one or more factors that need to be determined. The obvious explanation, i.e. a low transformation efficiency, can be ruled out, as the transformability of ML103 is comparable to MB001 and thus increased compared to the wildtype. Instead, this might be caused by an imbalanced Bxb1 activity, resulting in either not enough switching activity in *C. glutamicum* ML103, or, more likely, in too much Bxb1 being present. In the latter case, excess Bxb1 might bind to *attL* and *attR* and thereby act as an repressor of the switched promoter. This might be solved by modulating Bxb1 expression and will be addressed in the future.

IS*Cg1* in general was shown to be useable for the purpose of random mutagenesis. Analysis of the eight bp target site suggest a bias towards T for position three and four and towards A for the position five and six at the central four bp stretch with strong discrimination against G and C at positions four and 5 (Figure 4). While cursory inspection of the WebLogo implies that TTAA might be the predominant target motif, the list of tetranucleotide frequencies (Supplementary Table S4) reveals that this is not the case: neither is it the most common one nor is there any one sequence exceeding 20% of total transpositions with TTTA at 19.5% being the most prominent tetramer. In contrast to positions three to six, positions 1 and 2 as well as Position seven and eight show a slight tendency towards G and C. A distinct consensus for the target site preference however can not be derived. This strongly suggest that IS*Cg1* is applicable to target at such a random rate to potentially hit every feature in the genome of *C. glutamicum*. Indeed, the 3,207 independent sites hit in this work seem to be randomly distributed across the genome and displayed no general strand bias (Figure 3).

Thus, the transposon system using ML103 provides a useful, modular tool for random transposon mutagenesis with an easily applicable library generation protocol for ONT sequencing and IS*Cg1* as a randomly integrating transposase. The system can be adapted with little effort, e.g., by integration of an outward facing promoter on pML10 to allow for random gene activation/overexpression or by using a replicative vector to allow for multiple transpositions in a single mutant. For this purpose and to further facilitate industrial application, removal of the integrated kanamycin resistance cassette would be beneficial. This could be addressed by, e.g., combining our system with a Cre/*loxP*-mediated self-removal as described by Huang et al. (2017) by integrating two *lox* sites and an inducible Cre recombinase into the payload of pML10.

Taken together, the three strains *C. glutamicum* CR101, ML102, and ML103 in combination with plasmid ML103 provide a useful toolbox in basic research and industrial biotechnology.

## Data Availability

The datasets presented in this study can be found in online repositories. The names of the repository/repositories and accession number(s) can be found in the article/Supplementary Material.
