# Supplementary material for: Construction of an IS-Free Corynebacterium glutamicum ATCC 13 032 Chassis Strain and Random Mutagenesis Using the Endogenous ISCg1 Transposase
Source: Front Bioeng Biotechnol. 2021 Dec 15;9:751334. doi: 10.3389/fbioe.2021.751334 (PMC8715038; doi:10.3389/fbioe.2021.751334)
Supplement: Supplementary file 1 [file DataSheet1.pdf]

Supplementary Material

1 SUPPLEMENTARY TABLES AND FIGURES

Table S1: Oligonucleotides used in this study

Gene/Target	Name	[a] Sequence
pK18*mobsacB*	pK18_ga1	*GGTGCCTAATGAGTGAGCTA*
	pK18_ga2	*TAGCTCACTCATTAGGCACC*
	pk18_dgroel1	*TCTGATGTGGGGTGTT*CTGGGCTATCTGGACAAGGGAA
	pk18_dgroel2	*CAGCGTTGCGCCACA*TTTCCAGTCGGGAAACCTGTC
IS*Cg1a*_Bl	1a_d1	*TAGCTCACTCATTAGGCACC*-CGGGTGCTGGATTGTTCTTC
	1a_d4	*GCTTTCTACGTGTTCCGCTT*-AATTCGGTGCGCCTAAAGG
IS*Cg1b*	1b_d1	*TAGCTCACTCATTAGGCACC*-CGGGTTCCTCATATCCATCCAC
	1b_d2	CCACTTAAGAGCTCCAGTTGTGC
	1b_d3	*ACTGGAGCTCTTAAGTGG*ATTTTTAGGTCGGGATCAGAACAGC
	1b_d4	*GCTTTCTACGTGTTCCGCTT*-CAGACATCGCGAATGCTTGC
IS*Cg1c*_Bl	1c_d1	*TAGCTCACTCATTAGGCACC*-GCAACCGTAATCGCAGTTGG
	1c_d4	*GCTTTCTACGTGTTCCGCTT*-TAGCCAGGTCATCCATGAACG
IS*Cg1d*_Bl	1d_d1	*TAGCTCACTCATTAGGCACC*-CTAAACCCACAGTGCGAAGAC
	1d_d4	*GCTTTCTACGTGTTCCGCTT*-TTTCGCAGAGTCTTGGTGAGC
IS*Cg1e*	1e_d1	*TAGCTCACTCATTAGGCACC*ACCTCACCGCAATCACCG
	1e_d4	*GCTTTCTACGTGTTCCGCTT*-CTACAGCTTTCTCACCGCATCC
IS*Cg2b*_Bl	2b_d1	*TAGCTCACTCATTAGGCACC*ACCTGGCATGGACGAAAC
	2b_d4	*GCTTTCTACGTGTTCCGCTT*-TTATCAACGAGCGTGATCGTTC
IS*Cg2c*_Bf	2c_d1	*TAGCTCACTCATTAGGCACC*-CCAGACATGGATCTCGATTCG
	2c_d4	*GCTTTCTACGTGTTCCGCTT*-AAATGTCCCGAGCAACTTGG

Continued on next page

Table S1 – continued from previous page

Gene/Target	Name	^{a} Sequence
IS*Cg2d*_Bl	2d_d1	*TAGCTCACTCATTAGGCACC*-GGCACCGGATTTCGATCAGC
	2d_d4	*GCTTTCTACGTGTTCCGCTT*-CTCAGTGGCTCGTCGTATCG
IS*Cg2e*_Bl	2e_d1	*TAGCTCACTCATTAGGCACC*-GCTCAAGGACTTGTAGTGGAAGC
	2e_d4	*GCTTTCTACGTGTTCCGCTT*-TCAAGTTGAAGCGCCGCAAG
IS*Cg2f*_Bl	2f_d1	*TAGCTCACTCATTAGGCACC*-ATACGGACCTTCTGTGAGTGG
	2f_d4	*GCTTTCTACGTGTTCCGCTT*-TCccGCCAGTGTTCTTCTCTGC
IS*Cg3a*	3a_d1	*TAGCTCACTCATTAGGCACC*GCCGCAAACTGCTGTGG
	3a_d2	GGAGAGGACCATGCATTAG
	3a_d3	*CTAATGCATGGTCCTCTCC*CACGCCGGTCTTCCTAC
	3a_d4	*GCTTTCTACGTGTTCCGCTT*-CATCACTTGAACCATGCGTTCC
IS*Cg3b*_Bf	3b_d1	*TAGCTCACTCATTAGGCACC*-CCAGTCCACAACGATGCTGG
	3b_d4	*GCTTTCTACGTGTTCCGCTT*-CGTCAGTCAGACATGCATCC
IS*Cg4*_Bf	4_d1	*TAGCTCACTCATTAGGCACC*-CGGCCTTGATCTTGGCACC
	4_d4	*GCTTTCTACGTGTTCCGCTT*GCATTGTGCCTTGGTCGG
IS*Cg5a*_Bl	5a_d1	*TAGCTCACTCATTAGGCACC*-CGACCTGGAAAAACTCACCATC
	5a_d4	*GCTTTCTACGTGTTCCGCTT*-TGAAAAGGCGGTGGCGATTC
IS*Cg5b*_Bl	5b_d1	*TAGCTCACTCATTAGGCACC*-TCACCGTTATCGTGGTTGGC
	5b_d4	*GCTTTCTACGTGTTCCGCTT*-TTGATCCTACAGGTTTGCGC
IS*Cg5c*	5c_d1	*TAGCTCACTCATTAGGCACC*-GGCAGCTTCTGTAGCTTGC
	5c_d2	CACTGCTGTGGCTTTGTGTG
	5c_d3	*CACACAAAGCCACAGCAGTG*-GGATGGCCAACAAGATACTGG

Continued on next page

Table S1 – continued from previous page

Gene/Target	Name	[a] Sequence
	5c_d4	*GCTTTCTACGTGTTCCGCTT*-GCAGTTCCACTGGACCAATGG
IS*Cg6a*-IS*Cg7*	6a_d1	*TAGCTCACTCATTAGGCACC*-GGTGGTAGCCATAGCAGAAGC
	6a_d2	ACGGGCGCAGGGCATATC
	6a_d3	*GATATGCCCTGCGCCCGT*-TGACCGATGTCTCGACTCATC
	6a_d4	*GCTTTCTACGTGTTCCGCTT*-GAGTTCTTCATCTGCACGTCTG
IS*Cg6c*	6c_d1	*TAGCTCACTCATTAGGCACC*-ATTCAGCCTTAGGGCACCG
	6c_d2	TGCAGGGCCTTGTATTTCG
	6c_d3	*CGAAATACAAGGCCCTGCA*CGCCCGCATAGCAATAACC
	6c_d4	*GCTTTCTACGTGTTCCGCTT*-CCTGCGGTTCGGCAACAAC
IS*Cg8*	8_d1	*TAGCTCACTCATTAGGCACC*TGTCTCCACCAACTGAAG
	8_d2	AATCTTCGGGACCGTGAC
	8_d3	*GTCACGGTCCCGAAGATT*GGGATGTTAGGTGTCTAC
	8_d4	*GCTTTCTACGTGTTCCGCTT*-GCATGGGCTGCAGTTTCTATTC
IS*Cg9*	9_d1	*TAGCTCACTCATTAGGCACC*AATCGCATCGGCGACAAC
	9_d2	AACCGGAAGAGCCATCAC
	9_d3	*GTGATGGCTCTTCCGGTT*TTCAGACAGGGACAGG
	9_d4	*GCTTTCTACGTGTTCCGCTT*GGCTGGGACTAGAAACAC
IS*Cg12*	12_d1	*TAGCTCACTCATTAGGCACC*-TAGCAAGTGTGTCCGTCTGG
	12_d2	CACGAAAACGACGGGCGC
	12_d3	*GCGCCCGTCGTTTTCGTG*-GCATTTGTCTTTTCTAGCGATACC
	12_d4	*GCTTTCTACGTGTTCCGCTT*-TACCCATGATCTCACGAGCC
IS*Cg13a*-IS*Cg21a*_Bl	13a_d1	*TAGCTCACTCATTAGGCACC*-GCCACCGAAGCAGACATCC
	13a_d4	*GCTTTCTACGTGTTCCGCTT*-CGGTGCATTGGAATGGATCC
IS*Cg13b*_Bl	13b_d1	*TAGCTCACTCATTAGGCACC*-TGGCTGATGATGCTGCAGG

Continued on next page

Table S1 – continued from previous page

Gene/Target	Name	[a] Sequence
	13b_d4	*GCTTTCTACGTGTTCCGCTT*-GTCTTAAGCACCGGCAAGG
IS*Cg15a-b*_Cc	15_d1	*TAGCTCACTCATTAGGCACC*CACCTGCGCCATCTGAGG
	15_d4	*GCTTTCTACGTGTTCCGCTT*-AGAATTGCGGCAGCAGCTG
IS*Cg16a*_Cc	16a_d1	*TAGCTCACTCATTAGGCACC*ACCCGCTCCACCAACACC
	16a_d4	*GCTTTCTACGTGTTCCGCTT*-GACCCTTGCTGCATTCCTGC
IS*Cg16b*_Bl	16b_d1	*TAGCTCACTCATTAGGCACC*-GCAATAAGCACCTTCAGCCG
	16b_d4	*GCTTTCTACGTGTTCCGCTT*-GTGGAGCTTTCGTAGCCTTAGG
pCRn110d	pK18_bxbI_1	CCACGCCGGTCTTCCTAC
	pK18_bxbI_2	GAGAGGACCATGCATTAGCTGG
bxbI	bxbI_ins1	*GAAGACCGGCGTGG*TTGACAATTAATCATCCGGCTCGT-ATAATGAAAGAGGAGAAAATGCGCGCACTCGTT
	bxbI_ins2	*CTAATGCATGGTCCTCTC*TTAGGACATGCCGGTGTGCAG
groEL1	d1_groEL1	TGTGGCGCAACGCTGTATAT
	d2_groEL1	*GGTGTTTTTCACAGAACT*CTACTTCTCGACGATTGCGAGG
	d3_groEL1	AGTTCTGTGAAAAACACCGTGG
	d4_groEL1	AACACCCCACATCAGAGAACG
pUC19	switch_ga1	*TATTGGTGCCCTTAAACG*GACTCTAGAGGATCCCCGGG
	switch_ga2	*TGCACAGCCATACCACAG*GACCTGCAGGCATGCAAG
IS*Cg1*_IR_Kan	i1_IR_Kan_ga1	*GTCGTCGACAAGCCGGCCGA*-CTCGAGAAAGGAGGACAACCATGAAGTCTAC-CGGCAACATCATC
	i1_IR_Kan_ga2	CGTTTAAGGGCACCAATAACTGC
attP-attB-switch	attP_ga1	CTGTGGTATGGCTGTGCAGG
	attP_ga2	*CCAGACAAACCACGA*TGCCAGAACCGTTATGATGTCG
	attP_Pcons	TCGTGGTTTGTCTGGTCAACCACCGCGGTCT-CAGTGGTGTACGGTACAAACCCGCTAG-*CATTATACCTAGGACTGAGCTA*
	Pcons_attB	TCGGCCGGCTTGTCGACGACGGCGGTCTCC-GTCGTCAGGATCATCCGGGCTTGACAGC-*TAGCTCAGTCCTAGGTATAATG*
Adapter	D7_top	[P]-GATCGGAAGAGCACACGTCTG
	D7	CAGACGTGTGCTCTTCCGATCT

Continued on next page

Table S1 – continued from previous page

Gene/Target	Name	[a] Sequence
outer_tnp	tnp_o	[Bio]-CTTCAGTGACAACGTCGAGC
inner_tnp	tnp_i	[P]-AACGATCCTCATCCTGTC

[a] Bases in *italics* denote the overlapping regions used for Gibson Assembly.
[b] Postfix _Bl indicates "*B. lactofermentum*" as template, _Bf "*B. flavum*", and _Cc "*C.ċrenatum*".
[c] Prefix [P]- indicates phosphorylation, [Bio]- indicates biotinylation ".

Table S2. Plasmids used in this study

Name	Relevant genotype / information [a]	Source/reference
E. coli		
pK18*mobsacB*	*sacB*, *lacZ*α, Km^r, mcs	Schäfer et al., 1994
pCRn100d	pK18*mobsacB* carrying IS*Cg1a*_Bl_del	this study
pCRn101d	pK18*mobsacB* carrying IS*Cg1b*_del	this study
pCRn102d	pK18*mobsacB* carrying IS*Cg1c*_Bl_del	this study
pCRn103d	pK18*mobsacB* carrying IS*Cg1d*_Bl_del	this study
pCRn104d	pK18*mobsacB* carrying IS*Cg1e*_del	this study
pCRn105d	pK18*mobsacB* carrying IS*Cg2b*_Bl_del	this study
pCRn106d	pK18*mobsacB* carrying IS*Cg2c*_Bf_del	this study
pCRn107d	pK18*mobsacB* carrying IS*Cg2d*_Bl_del	this study
pCRn108d	pK18*mobsacB* carrying IS*Cg2e*_Bl_del	this study
pCRn109d	pK18*mobsacB* carrying IS*Cg2f*_Bl_del	this study
pCRn110d	pK18*mobsacB* carrying IS*Cg3a*_del	this study
pCRn111d	pK18*mobsacB* carrying IS*Cg3b*_Bf_del	this study
pCRn112d	pK18*mobsacB* carrying IS*Cg4*_Bf_del	this study
pCRn113d	pK18*mobsacB* carrying IS*Cg5a*_Bl_del	this study
pCRn114d	pK18*mobsacB* carrying IS*Cg5b*_Bl_del	this study
pCRn115d	pK18*mobsacB* carrying IS*Cg5c-19a*_Cc_del	this study
pCRn116d	pK18*mobsacB* carrying IS*Cg6a-7*_del	this study
pCRn117d	pK18*mobsacB* carrying IS*Cg6c*_del	this study
pCRn118d	pK18*mobsacB* carrying IS*Cg8*_del	this study
pCRn119d	pK18*mobsacB* carrying IS*Cg9*_del	this study
pCRn120d	pK18*mobsacB* carrying IS*Cg12*_del	this study
pCRn121d	pK18*mobsacB* carrying IS*Cg13a-21*_Bl_del	this study
pCRn122d	pK18*mobsacB* carrying IS*Cg13b*_Bl_del	this study
pCRn123d	pK18*mobsacB* carrying IS*Cg15a-b*_Cc_del	this study
pCRn124d	pK18*mobsacB* carrying IS*Cg16a*_Cc_del	this study
pCRn125d	pK18*mobsacB* carrying IS*Cg16b*_Bl_del	this study
pMLi002	pCRn110d carrying *bxbI*_int	this study
pMLd009	pK18*mobsacB* carrying *groEL*del	this study
pUC19	*lacZ*α, Amp^r, msc	Yanisch-Perron et al., 1985
pML10	pUC19 carrying IS*Cg1*_IR_Kan, attP-attB-switch	this study

[a] r superscript indicates resistance. Nx, Nalidixic acid; Km, Kanamycin; Amp, Ampicillin
[b] the postfix _del indicates inserts used for targeted gene deletion; _int indicates inserts used for targeted gene insertion.

Table S3. Growth rates in BHI medium

Strain	Growth rate [a]
MB001	0.382 ± 0.016
CR101	0.387 ± 0.015
ML102	0.379 ± 0.014
ML103	0.384 ± 0.003

[a] Calculated from 4 independent biological replicates with 4 technical replicates sampled every 15 minutes, using the BioLection software a sliding window over an 2 hour interval (i. e. 8 data points). Values denote the averages of the growth rates of the biological replicates ± the standard deviation of of those averages.

Table S4. Frequencies of the 10 most abundant central tetranucleotide sequences in the target site of 2422 ISCg1 transposition sites with more than one mapped read

central 4 bp of target site	(+) strand	(-) strand	total	fraction [%]
TTTA	256	217	473	19.5
TTAA	201	221	422	17.4
TAAA	160	180	340	14.0
TTTG	69	78	147	6.1
ATTA	50	73	123	5.1
ATAA	58	43	101	4.2
TTAT	50	51	101	4.2
TAAT	43	50	93	3.8
CAAA	52	36	88	3.6
TTTT	39	30	69	2.8

1.1 Figures

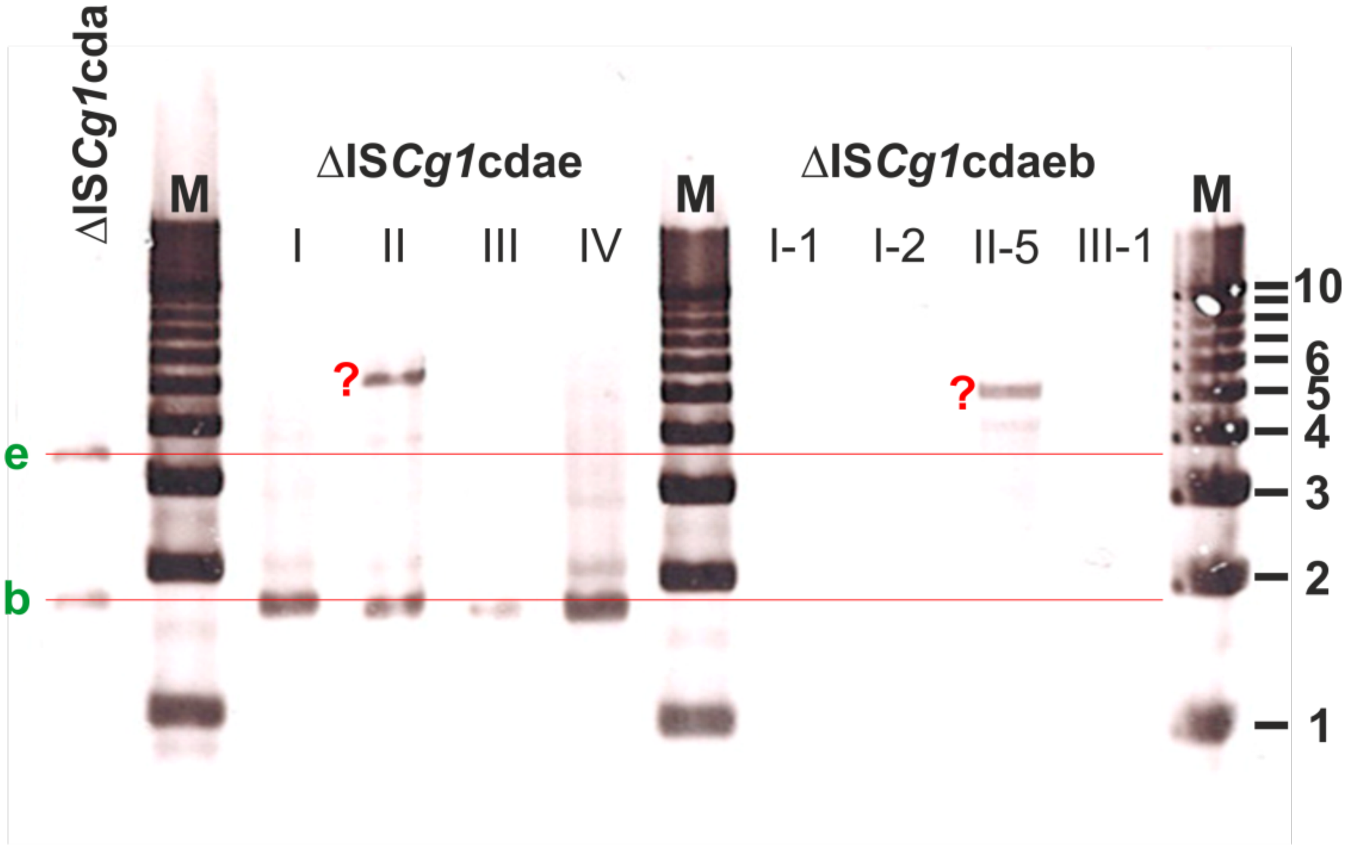

Figure S1. Southern blot and hybridization to track IS*Cg1* copies during IS*Cg1* removal. Genomic DNA was isolated from several different ΔIS*Cg1cda* strains with a potential IS*Cg1e* deletion respectively several different ΔIS*Cg1cdae* strains with a potential IS*Cg1b* deletion, cut with *Nhe*I, *Sal*I, and *Bgl*II. Genomic DNA from a validated ΔIS*Cg1cda* mutant was used as a control. After Southern blotting, a DIG-labelled IS*Cg1* probe was created, hybridized and visualized using the DIG DNA-Labelling- and Detection Kit (Roche, Germany). Red lines indicate the potential positions of IS*Cg1e* and IS*Cg1b*, respectively. Red question marks indicate the presence of potential IS*Cg1* transposition events.

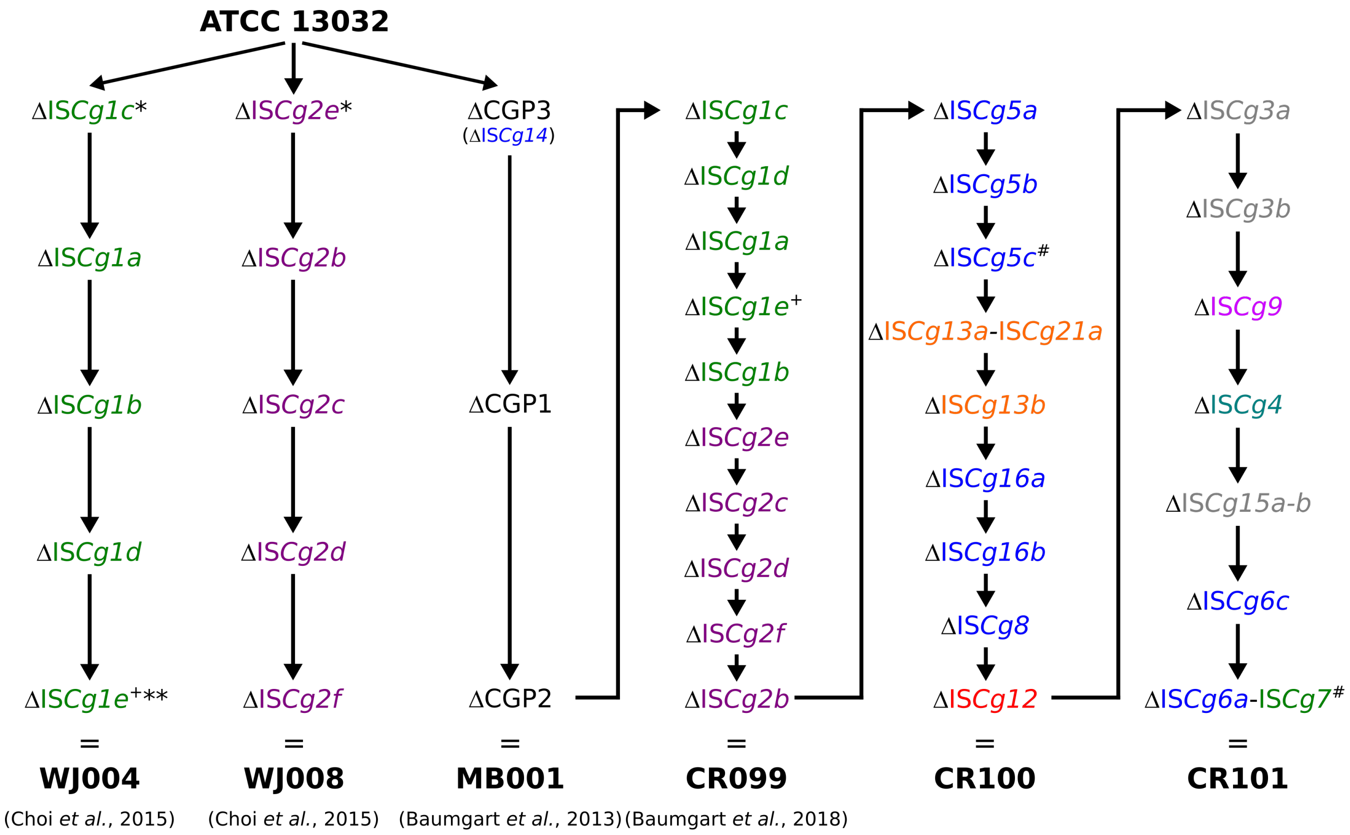

Figure S2. Overview of the *C. glutamicum* strains created/used by Choi et al. (2015); Baumgart et al. (2013, 2018), and this study with systematic targeted deletions of mobile genetic elements. Colors denote different IS families: IS*L30*, IS*3*, IS*30*, IS*4*, IS*256*, IS*110*, IS*6*, and IS*5*, respectively.

* indicates IS elements found to be absent by Choi et al. (2015) in their version of *C. glutamicum* ATCC 13032.

+ denotes IS*Cg1* elements not reported to be present in ATCC 13032.

# marks potentially inactive (partial) IS elements.

** It is important to note that IS*Cg1e* reported by Choi et al. (2015) (position 613,777) differs from that found in MB001 (position 541,126).

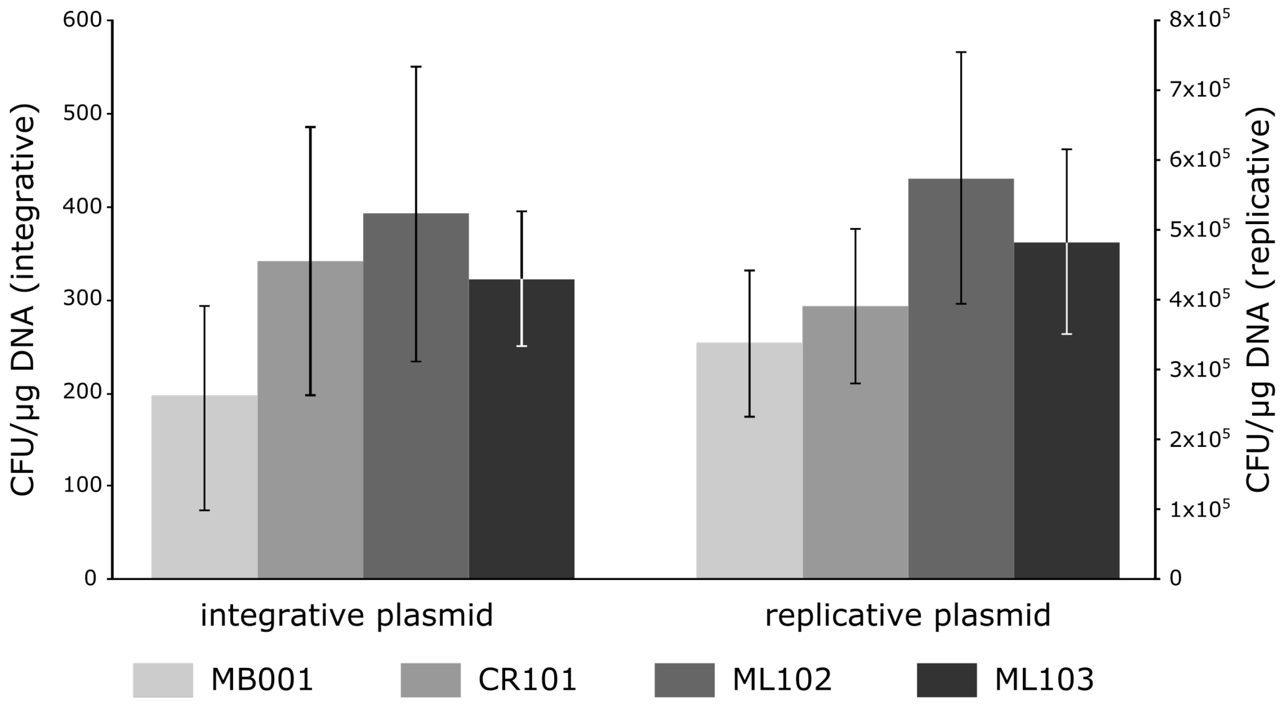

Figure S3. Transformation efficiency of *C. glutamicum* strains MB001, CR101, ML102, ML103. 100 μl cell suspension containing 10^9 cells was mixed on ice with 500 ng (replicative) respectively 1,000 ng (integrative) plasmid DNA. After regeneration, aliquots were plated on solid BHI medium containing 25 mg per liter kanamycin. CFU were counted after 2 days incubation at 30 °C and the number of transformants was calculated as CFU per μg DNA. Bars represent the means of the biological replicates, error bars denote the standard deviations.

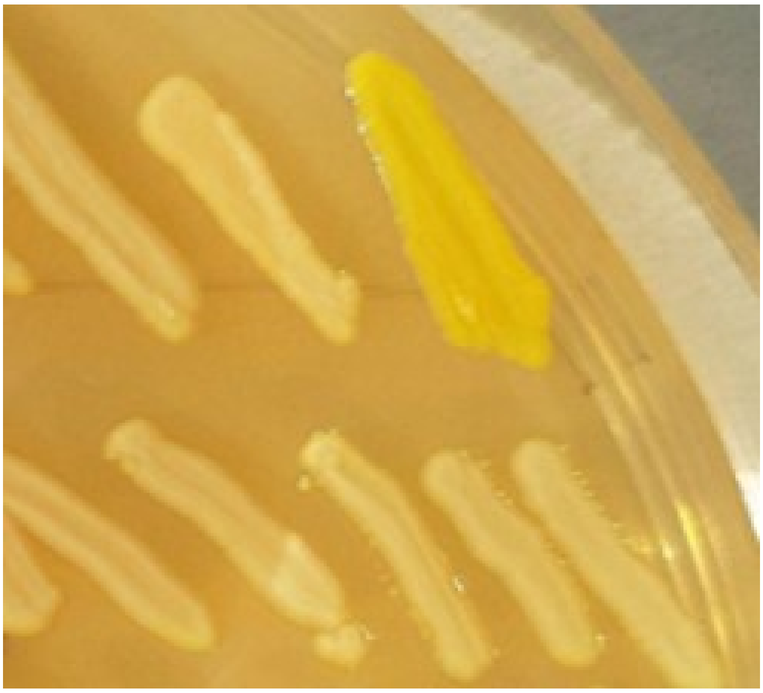

Figure S4. A phenotypically noticeable mutant with transposition in *crtR* (Henke et al., 2017) on agar plate (rightmost colony).

REFERENCES

Baumgart, M., Unthan, S., Kloß, R., Radek, A., Polen, T., Tenhaef, N., et al. (2018). *Corynebacterium glutamicum* chassis C1*: Building and testing a novel platform host for synthetic biology and industrial biotechnology. *ACS Synthetic Biology* 7, 132–144. doi:10.1021/acssynbio.7b00261

Baumgart, M., Unthan, S., Rückert, C., Sivalingam, J., Grünberger, A., Kalinowski, J., et al. (2013). Construction of a prophage-free variant of *Corynebacterium glutamicum* ATCC 13032 for use as a platform strain for basic research and industrial biotechnology. *Applied and Environmental Microbiology* 79, 6006–6015. doi:10.1128/aem.01634-13

Choi, J. W., Yim, S. S., Kim, M. J., and Jeong, K. J. (2015). Enhanced production of recombinant proteins with *Corynebacterium glutamicum* by deletion of insertion sequences (IS elements). *Microbial Cell Factories* 14. doi:10.1186/s12934-015-0401-7

Henke, N. A., Heider, S. A. E., Hannibal, S., Wendisch, V. F., and Peters-Wendisch, P. (2017). Isoprenoid pyrophosphate-dependent transcriptional regulation of carotenogenesis in *Corynebacterium glutamicum*. *Frontiers in Microbiology* 8. doi:10.3389/fmicb.2017.00633

Schäfer, A., Tauch, A., Jäger, W., Kalinowski, J., Thierbach, G., and Pühler, A. (1994). Small mobilizable multi-purpose cloning vectors derived from the *Escherichia coli* plasmids pK18 and pK19: Selection of defined deletions in the chromosome of *Corynebacterium glutamicum*. *Gene* 145, 69–73

Yanisch-Perron, C., Vieira, J., and Messing, J. (1985). Improved M13 phage cloning vectors and host strains: nucleotide sequences of the M13mpl8 and pUC19 vectors. *Gene* 33, 103–119. doi:10.1016/0378-1119(85)90120-9
